# Commentary: The ETP-based APC resistance assay remains the gold standard for evaluating the APC resistance phenotype in users of combined oral contraceptives

**DOI:** 10.3389/fcvm.2025.1611404

**Published:** 2025-07-15

**Authors:** Laure Morimont, Jonathan Douxfils

**Affiliations:** ^1^ Qualiblood sa, QualiResearch, Liège, Belgium; ^2^ Clinical Pharmacology and Toxicology Research Unit, Namur Research Institute for Life Sciences, University of Namur, Namur, Belgium; ^3^ Hematology Department, Centre Hospitalier Universitaire Clermont-Ferrand, Hopital Estaing, Clermont-Ferrand, France

**Keywords:** endogenous thrombin potential (ETP), combined oral contraceptives (COC), activated protein C resistance, ETP-based APC resistance assay, thrombomodulin (TM), activated protein C (APC)

A Commentary on Thrombomodulin is a stronger indicator of combined oral contraceptives-induced activated protein C pathway resistance in the thrombin generation test than activated protein C By Ninivaggi M, Sokolova L, Donkervoort D, de Laat B and de Laat-Kremers R (2024). Front. Cardiovasc. Med. 11:1490601. doi: 10.3389/fcvm.2024.1490601

To the Editor,

We read with great interest the article of Ninivaggi et al. on activated protein C (APC) resistance induced by combined oral contraceptives (COCs) and would like to express our concerns regarding the ability of the thrombomodulin (TM)-based assay to more effectively discriminate between COC users and non-users.

The authors stated that the type of progestin in COCs modulates the associated risk of thrombosis, suggesting a higher risk of venous thromboembolism (VTE) with third-generation COC containing desogestrel compared with second-generation COC containing levonorgestrel. However, the actual risk depends primarily on the dose of ethinylestradiol (EE). For third-generation COCs, the VTE risk increases from 3.4 (95% CI: 2.5–4.6) to 4.3 (95% CI: 3.3–5.6) when EE dosage increases from 20 to 30 µg, while the dose of desogestrel remains constant. In contrast, for second-generation COCs, the VTE risk remains stable regardless of EE dose—2.2 (95% CI: 1.3–3.6) for EE 20 µg and 2.4 (95% CI: 1.8–3.2) for EE 30 µg—because EE and levonorgestrel doses increase proportionally (1). These long-established epidemiological data clearly show that the estrogenic component is the primary determinant of VTE risk, while the associated progestin modulates it to varying degrees. This debate is reminiscent of the misuse of the “generation” term used following chronological order of appearance on the market and not based on any pharmacological basis (2). Ignoring these differences leads to the assumption that all COCs confer the same VTE risk, which is certainly not the case. For instance, depending on the patient's coagulable status, a natural estrogen-based COC (which demonstrated a lower risk of VTE) may be appropriate, whereas an EE-based COC would not be (3).

This study aimed to evaluate whether adding TM or APC to the thrombin generation (TG) assay results in differing sensitivity for detecting APC resistance in COC users. This rationale appears to stem from our 2021 review in *Frontiers in Endocrinology* (4), which raised considerations about the use of TM, APC, or both in assessing APC resistance. However, this interpretation is inaccurate, as our review does not discuss the methodology of the endogenous thrombin potential (ETP)-based APC resistance assay. Rather, it highlights the relevance of using a global sensitivity assay like the ETP-based APC resistance assay to determine a patient's coagulation status and support clinical decision-making when prescribing contraceptives.

Briefly, the ETP-based APC resistance assay is based on the measurement of thrombin generation in the presence and absence of a defined amount of exogenous APC. The endpoint of the test, which is the total amount of thrombin that has been generated over time, is quantitated by the ETP, which corresponds to the area under the thrombin generation curve. Results can be expressed as a percentage of inhibition, which reflects the decrease of the ETP parameter between the two conditions, with and without APC. In other words, when targeting 90% inhibition with a reference plasma, this means that the ETP(+APC) is reduced by 90% compared with the ETP(-APC).

The authors assert that TM is a better discriminator, but their results suggest otherwise. At the highest TM and APC concentrations—intended to achieve 90% ETP inhibition in healthy individuals—the observed mean inhibition was 86% for TM and only 73% for APC in non-users. This discrepancy highlights methodological limitations, especially regarding APC, as inhibition values near 90% were expected. The observed 73% inhibition with 5.5 nM APC suggests suboptimal dosing, which may reduce assay sensitivity. This could stem from their choice of pooled normal plasma to determine optimal TM and APC concentrations. Notably, the gender ratio of the pooled plasma is not reported, even though APC resistance levels vary between males and females, thereby influencing the assay's sensitivity.

Despite this limitation, the absolute difference in inhibition % between non-users and COC users was higher using 5.5 nM APC than 15 nM TM (17% vs. 9%). Even at the lowest tested concentrations (1.5 nM APC vs. 2 nM TM), the difference remained higher for APC (21% vs. 14%). These findings are evident in Figure 3D, E, and F of the original article. Moreover, Figure 3F demonstrates notable variability among COC users at 5.5 nM APC, potentially due to the absence of subgroup classification (i.e., EE-levonorgestrel, EE20-desogestrel, EE30-desogestrel). With 15 nM TM, this heterogeneity vanishes due to assay saturation, resulting in lower sensitivity to COC-induced changes.

The targeted 90% inhibition in a reference population is not chosen arbitrarily. By aiming for 90% inhibition, the assay operates near the plateau phase of the curve (*x*-axis, APC concentration; *y*-axis, inhibition%) (5), which helps minimize analytical variability. Indeed, variations in APC concentration will have a limited impact on the inhibition %, as the slope of the curve is flatter. Conversely, targeting 50% inhibition corresponds to the steepest part of the curve, where even minor variation in APC activity can result in significant changes in the inhibition %. From a clinical perspective, targeting 90% inhibition in a normal population allows for a greater sensitivity to the various estroprogestin associations, since the level of APC resistance can fluctuate across a range from 0% to 90% whereas this range is halved when aiming for 50% inhibition. Ultimately, targeting 90% inhibition provides greater analytical precision and enhanced clinical sensitivity.

For now, there is no available commercial kit for the intended purpose of assessing the thrombotic risk of women using COC. Solely, the methodology for the ETP-based APC resistance assay has been validated and standardized according to FDA and ICH guidelines. In this validated methodology, reagents (STG ThromboScreen®, Thrombin calibrator®, FluCa Kit®)—including APC originally from Enzyme Research Laboratory—are supplied by Diagnostica Stago, ensuring reliable and consistent availability. Indeed, an important aspect of a validated technology is the ability of providing reproducible results over time. In other words, batch changes must be properly controlled and fully documented. The use of home-made methodologies, as performed by Ninivaggi et al. fails to meet the standard of analytical rigor as outlined above.

Over the past 30 years, many studies have explored the effects of COC on hemostasis. Regarding the protein C-protein S anticoagulant pathway, increases in protein C and decreases in protein S levels have been described (6). When exogenous TM is used, the patient endogenous protein C is activated and then associates with endogenous protein S to inactivate FVa and then FVIIIa. In women using COC, the decrease of protein S could be, to some extent, counterbalanced by the increase in protein C, leading to a lower TM-induced APC resistance. Additionally, as the authors note, TM engages multiple upstream coagulation pathways, leading to broader interferences and attenuated assay sensitivity. In contrast, when exogenous APC is used, protein S is the primary limiting factor, yielding greater sensitivity to COC-induced changes.

The conclusions drawn in this study should be interpreted with caution. The results do not convincingly support the claim that TM is a better discriminator than APC. To date, the ETP-based APC resistance assay remains the only method endorsed in EMA guidelines for the assessment of new steroid contraceptives in women. In addition, the plasma coagulation inhibitor subcommittee of the International Society on Thrombosis and Haemostasis Scientific and Standardisation Committee stated that this test, specifically, should be the phenotypic test of choice for assessing acquired APC resistance induced by COC (7).
